# Medical Treatment for Endometriosis: One Size Does Not Fit All

**DOI:** 10.3390/jcm15062408

**Published:** 2026-03-21

**Authors:** Paolo Vercellini, Noemi Salmeri, Veronica Bandini, Beatrice Conca, Paola Viganò, Edgardo Somigliana, Michele Vignali

**Affiliations:** 1 Academic Centre for Research on Adenomyosis and Endometriosis, Department of Clinical Sciences and Community Health, Università degli Studi, 20122 Milan, Italy; noemi.salmeri@unimi.it (N.S.); veronica.bandini@unimi.it (V.B.); beatrice.conca@unimi.it (B.C.); paola.vigano@policlinico.mi.it (P.V.); edgardo.somigliana@unimi.it (E.S.); michele.vignali@unimi.it (M.V.); 2 Fondazione IRCCS Ca’ Granda Ospedale Maggiore Policlinico, Via Commenda 12, 20122 Milan, Italy

**Keywords:** endometriosis, adenomyosis, pelvic pain, dysmenorrhea, dyspareunia, progestogens, estrogen-progestogens, GnRH analogs

## Abstract

Endometriosis is associated with nociceptive pain, as well as peripheral and central sensitization. Evidence-based treatment suggestions for controlling endometriosis should be based on the convergence of the best scientific evidence, physicians’ clinical expertise, and the values and priorities of individual patients. In this non-systematic, comprehensive narrative review, data from available randomized controlled trials and meta-analyses on hormonal treatment for symptomatic endometriosis are interpreted through the lens of clinical experience. The role of patients in defining therapeutic trade-off balances is also taken into consideration. Most symptomatic patients benefit from hormonal therapy, including first-line (progestogens and estrogen-progestogen combinations) and second-line (GnRH agonists and antagonists) medications, to relieve nociceptive pain. To reduce the risk of venous and arterial thrombosis and avoid stimulating lesions, it is preferable to use combinations containing body-identical estrogens rather than ethinyl-estradiol. The main adverse effect of first-line medications is irregular bleeding, which adversely impacts efficacy, tolerability, and adherence. If progestogens and estrogen-progestogens do not improve health-related quality of life (HRQoL), promptly stepping up to GnRH analogues combined with add-back therapy is indicated. Add-on rather than upfront combination therapy is suggested. Separating the analogues and add-back therapy allows for choosing the compounds that best suit the characteristics of individual patients. Transdermal body-identical estradiol use is proposed in combination with both progestogens and GnRH analogues. Similar satisfactory outcomes are achieved with GnRH agonists and antagonists. Evidence on the use of neuromodulatory drugs to treat neuropathic and nociplastic pain is derived from studies of other chronic pain conditions and shows limited effectiveness. The two mainstays of hormonal therapy are (i) ovariostasis and (ii) amenorrhea. “Medical treatment failure” should not be declared unless a shift from first-line to second-line medications has been undertaken whenever these conditions are not met. For severely symptomatic adolescents and young women, secondary prevention through ovariostasis and amenorrhea should be pursued promptly to improve HRQoL, halt lesion progression, and preserve reproductive potential.

## 1. Introduction: What This Review Is and Is Not

“*Evidence-based and experience-based medicine have their own limitations and are not mutually exclusive but rather complementary for sound clinical practice*”

(Ata and Saridogan [1])

“*Notwithstanding important achievements, the clinical impact of EBM has remained limited, especially in surgery and in endometriosis management*”

(Koninckx et al. [2])

Several excellent reviews on the management of symptomatic endometriosis are available that provide factual information and quantitative estimates of the efficacy of different medications based on the best available evidence [3,4,5,6,7,8,9,10,11,12,13,14]. Thus, it is doubtful whether another review could substantially add new information that would be useful for patients and healthcare providers.

Randomized controlled trials (RCTs) are considered the best possible modality for estimating the magnitude of the effect of medical interventions. However, the findings may not be generalizable for several reasons, including study populations with specific characteristics, the selection of outcomes that do not align with those most important to patients, the additional effect of being included in a trial, and the particularly favorable research setting that cannot be replicated in everyday practice [2,15,16].

As early as 60 years ago, Austin Bradford Hill noted that data from RCTs might not permit the customization of treatments based on individual patients’ needs and priorities. Furthermore, he emphasized the importance of integrating experimentation with clinical observation and critical thinking in medical research [17].

In recent years, there has been an exponential growth in the number of published systematic reviews and meta-analyses. However, due to several limitations and drawbacks, it is questionable whether this phenomenon necessarily translates into better care. Indeed, the results of meta-analyses are often scarcely reliable or conflicting [18,19,20,21,22]. Moreover, even when methodologically impeccable, available systematic reviews, meta-analyses, and network meta-analyses may convey messages that are not entirely aligned with clinical experience [16,23,24]. Experts in hormonal medications can contextualize these findings and resolve inconsistencies. However, those who are unfamiliar with the effects of various drugs may be given information that, when applied in practice, may not always benefit patients.

For these reasons, we will interpret and apply data from RCTs and meta-analyses through the lens of clinical experience. Additionally, we will take into consideration the role of patients in defining trade-off balances. In other words, we will try to offer evidence-based practice suggestions resulting from the convergence of the current best scientific evidence, physicians’ clinical expertise, and individual patients’ values and priorities. These three factors are key to determining shared decisions.

Not all available medications will be considered or will be described in detail. Priority will be given to those compounds that can provide adequate pain relief and have the best possible safety profile. Indeed, medical treatment for severely symptomatic endometriosis is intended for years, if not decades. Therefore, safety is as important as efficacy. Moreover, therapies should be well-tolerated because both the disease and the chosen treatment can negatively impact health-related quality of life (HRQoL). The relationship between tolerability and adherence is critical to the effectiveness of any therapy. Finally, when not correctly indicated, expensive medications consume healthcare resources unnecessarily. This has implications at the individual and community levels, so it is ethical to selectively prescribe more costly compounds when less expensive ones are ineffective, not tolerated, or contraindicated.

Only progestogens supported by adequate available evidence regarding their effect on women with endometriosis-associated pain will be described. For the first time, we will not consider all combined oral contraceptives (COCs), but rather, only those containing a body-identical estrogen with a low risk of venous thromboembolism (VTE). Specifically, COCs containing ethinyl-estradiol (EE), a strong inducer of hepatic procoagulant factors, will not be addressed [25,26,27]. Additionally, only monophasic COCs will be included, because multiphasic ones do not allow for stable estrogen and progestogen serum levels, which are essential for successfully treating endometriosis with hormones. Aromatase inhibitors have also been excluded because they provide limited additional benefit when combined with first-line medications in premenopausal patients and are associated with subjective (arthralgia) and objective (reduced bone mineral density) adverse effects, especially when combined with GnRH analogues. Furthermore, costs increase, and the overall therapeutic balance remains unclear [28].

According to the current view on coexistence of different types of pain in endometriosis patients [8,12,29,30,31], we have also synthesized the limited available evidence on those medications that could impact pain deriving from peripheral and central sensitization. These medications could be combined with hormonal compounds that target lesion-related nociceptive pain. However, our review of this specific topic is brief and merely indicative.

We will not discuss common analgesics, such as non-steroidal anti-inflammatory drugs (NSAIDs). According to a Cochrane review, the effectiveness of naproxen and other NSAIDs in managing pain caused by endometriosis is unclear [32]. Nonresponse to NSAIDs is often associated with the presence of adenomyosis and endometriosis [33]. In addition, a synergistic thrombogenic effect of COCs and NSAIDs has been observed [34]. This is clinically relevant as both types of drugs are frequently used together in patients with symptomatic endometriosis. Cannabinoids, dietary supplements, probiotics, minerals, multivitamins, herbal products, and compounds that have not yet undergone phase III trials will also not be addressed here. The same applies to the application of botulinum toxin to the female pelvic floor for controlling painful symptoms, including dyspareunia. However, evidence of its efficacy is accumulating [35], and data from a randomized, double-masked, phase 2 trial were recently published [36]. Thus, this treatment modality could be considered alongside hormonal therapy for patients with endometriosis and pelvic floor muscle spasm.

The reader may refer to available reviews for more information (e.g., [37]) on overlapping chronic pain conditions (COPCs) that frequently coexist with endometriosis, such as vulvodynia/vestibulodynia, interstitial cystitis/painful bladder syndrome, irritable bowel syndrome, chronic low back pain, chronic tension or migraine headaches, temporomandibular disorders, fibromyalgia, and myalgic encephalomyelitis/chronic fatigue syndrome.

Our suggestions generally align with the ESHRE guideline [38], the updated NICE guideline [39], the SOGC guideline [40], and the ACOG guideline [41] for managing endometriosis and chronic pelvic pain (CPP). However, we acknowledge that the recommendations outlined in these guidelines have sometimes been interpreted and adapted with the hope of facilitating healthcare providers and benefiting patients by providing easily implementable, practical suggestions. Thus, this experience-driven review may differ from the above guidelines in several aspects.

However, this may occasionally tip the balance in favor of expert opinion, which is the lowest level of the hierarchy of medical evidence [2,15]. Additionally, readers should be aware that, despite a thorough PubMed search prioritizing systematic review with meta-analyses, RCTs, and seminal articles published within the last decade, this approach is subject to a high risk of confirmation bias due to publication cherry-picking. Therefore, this non-systematic, narrative review should be intended exclusively as a general framework for pragmatic considerations and practical suggestions that could be used after consulting more reliable sources of scientific information.

## 2. The Mainstays of Hormonal Treatment for Adenomyosis and Endometriosis: (I) Ovariostasis and (II) Amenorrhea

Several investigators from various fields, including gynecology, epidemiology, endocrinology, evolutionary biology, and public health, consider the current post-industrial menstrual pattern to be unphysiological. Repetitive ovulatory menstruations for decades, without prolonged periods of amenorrhea due to pregnancies and lactation, may be an important contributing cause of some disorders of the female genital system, such as ovarian, endometrial, and breast cancers, as well as adenomyosis and endometriosis [42,43,44,45,46,47,48].

Moreover, Leyendecker, Kunz, and colleagues highlighted already in the nineties that the association between adenomyosis and endometriosis is so robust as to suggest causation [49,50,51,52]. Specifically, adenomyosis would develop first, followed by endometriosis due to augmented retrograde menstruation and transtubal reflux of basal endometrial cells. This would be secondary to myometrial inflammation and fibrosis, which leads to dysfunction with altered cervical-fundal peristalsis during menses (see, for a synthesis, [53,54]. Unfortunately, adenomyosis remains underdiagnosed [4,55,56].

Brosens et al. also considered adenomyosis and endometriosis so closely related that they described an “endo-myometrial dysfunction syndrome” [57]. Accordingly, Exacoustos et al. proposed using ultrasonography (US) to evaluate the thickness and alterations of the junctional zone, as a proxy for adenomyosis, to presumptively diagnose superficial peritoneal endometriosis [58]. Bulun et al. define adenomyosis and endometriosis as “sister entities” with an identical pathogenesis as they demonstrably originate from the same cellular oligoclones [59]. Leyendecker et al. stated that “the aetiology of endometriosis is primarily the aetiology of adenomyosis” [51].

Here, we suggest taking it a step further by proposing that hormonal therapy for endometriosis is primarily hormonal therapy for adenomyosis. The typical symptoms of adenomyosis, i.e., dysmenorrhea, menorrhagia, and, to a lesser extent, dyspareunia, are extremely common in patients with endometriosis. It is difficult to determine which condition causes which symptoms. In fact, when pelvic symptoms persist after conservative surgery for endometriosis, adenomyosis is often present and perpetuates pain [29,60]. Fortunately, the treatment for endometriosis remains the same when it coexists with adenomyosis [61,62] and is based on two key therapeutic principles: (i) reducing local and systemic levels of 17β-estradiol (E2) via ovulation inhibition, and (ii) preventing uterine and pelvic bleeding via menstrual suppression.

(i) Repetitive ovulations result in increased estrogenic exposure, both locally in the pelvis and systemically via elevated serum E2 levels. Local estrogen overexposure can favor implantation, survival, and proliferation of refluxed endometrial cells [59,63,64]. Moreover, excessive and/or chronic exposure to E2 is pro-inflammatory [65,66,67] and may aggravate endometriosis-associated pain [64,68]. At high-amplitude fluctuating serum concentrations, E2 can sensitize nociceptors, elevate pain perception [69], exacerbate uterine pain, and produce stimulus-intensity-dependent increases in inflammation-evoked nociceptive behavior in a mouse model [70,71]. Local excess of E2 favors macrophage-nerve interactions [4], and systemic cumulative estrogenic overexposure may promote neuroinflammation [72,73]. These phenomena may contribute to the onset of central sensitization, persistent pelvic pain, and cross-organ pain amplification [4].

Based on indirect evidence from different research groups, Crespi and Evans not only confirm that endometriosis is associated with a high pre- and postnatal E2-to-testosterone ratio. They also hypothesize that this imbalance favors a proinflammatory condition and results in lower levels of β-endorphin in the central nervous system (CNS). Thus, they propose “a centrally mediated effect beginning in early prenatal development, and persisting through adult life, with notable effects on pain sensitivity” [74]. Direct evidence of such a hormonal imbalance is now available [75].

(ii) According to Guo, “bleeding is a hallmark of vascular injury and thus tissue injury. In all organisms, following a tissue injury, the evolutionarily conserved tissue repair program will immediately kick in. In other words, adenomyotic lesions thus resemble wounds that undergo repeated tissue injury and repair (ReTIAR). Consequently, they would experience the well-known four phases in tissue repair: hemostasis, inflammation, proliferation, and remodeling” [76]. Thus, hemostasis is evolutionarily favorable because it protects against potentially fatal hemorrhages and infections. However, it also triggers platelet activation and engages different types of immune cells. The acute inflammatory process that occurs during menstruation leads to tissue remodeling and fibrosis when repeated. This process is similar in both the uterine and pelvic cavities. In the uterus, it results in adenomyosis, myometrial dysfunction, heavy menstrual bleeding, and dysmenorrhea. In the pelvis, it leads to the so-called deep infiltrating lesions, which frequently cause deep dyspareunia and dyschezia [76,77,78,79]. Notably, the amount of retrograde menstrual flow detected by ultrasound is directly proportional to the amount of orthograde menstrual flow [80]. Patients with adenomyosis and endometriosis experience heavy menstrual bleeding and a high amount of retrograde menstruation significantly more frequently than women without these conditions [80].

Given this background, any hormonal therapy should be tailored to achieve a stable and mildly hypoestrogenic environment through “ovariostasis” [48]. Anovulation and amenorrhea counteract both the endocrine and the inflammatory pain components resulting from recurrent ovulation and repetitive menstruation, respectively. This reduces pelvic and systemic inflammation, potentially improving both nociceptive and non-nociceptive pain. Moreover, this pharmacological approach can prevent the progression of both adenomyosis and endometriosis, thus acting as an effective secondary preventive measure [55,81,82,83,84].

## 3. First-Line Hormonal Drugs for Nociceptive Pain

Progestogen monotherapies and estrogen-progestogen combinations represent first-line medication used to relieve endometriosis-associated pain symptoms [38,39]. For more information on the specific endocrine characteristics and relative potency of various progestogens and estrogens, the readers should refer to published reviews such as those by Stanczyk et al. [85,86], Hapgood et al. [87], and Żyła et al. [88].

1. Progestogen monotherapies: Selection based on data for endometriosis treatment

Despite the convincing arguments about the excessive estrogenic stimulus exerted by most available COCs and suggestions to prefer progestogens when treating patients with endometriosis [89], a meta-analysis of RCTs comparing progestogens and COCs did not demonstrate significant between-group differences in pain symptom relief or adverse effects associated with the two medication classes [90]. Still, several experts consider progestogens to be the reference standard first-line treatment for symptomatic endometriosis [89]. For an overview of progestogens’ effects on pain symptoms associated with the disease, the reader can refer to the recent Cochrane review [91] and to comprehensive reviews on this drug category (e.g., [92]). Here, we will address only those progestogens supported by a substantial body of evidence: dienogest (DNG), norethisterone acetate (NETA) used at a dose of 2.5 mg a day, and levonorgestrel delivered via intrauterine device (LNG-IUD). Additionally, we will summarize the limited data regarding drospirenone (DRSP), a compound with a purportedly favorable tolerability profile. We will not address medroxyprogesterone acetate (MPA), in either oral or depot formulations, because of its potential thromboembolic risk and detrimental effect on bone mineral content [34,93,94]. The use of subdermal implants will not be addressed either. A recent systematic review with meta-analysis, including six RCTs and 1503 participants, demonstrated that subdermal implants were associated with a significantly and substantially higher risk of acne, weight gain, treatment dissatisfaction, and eventual device removal due to side effects, including irregular bleeding, compared to LNG-IUD [95].

a. Dienogest

Dienogest is the most studied progestogen for treating symptomatic endometriosis. It is currently the reference standard among first-line hormonal medications used to relieve chronic pain symptoms associated with the disease. It combines satisfactory efficacy and tolerability, including adequate bleeding control, and is safe and affordable since generic unbranded preparations became available. Here, we synthesize the results of systematic reviews and meta-analyses published on DNG since 2020.

Wu et al. [96] evaluated the effect of oral DNG, 2 mg/day, in symptomatic patients with deep infiltrating endometriosis (DIE). After pooling the data from five studies, comprising 256 participants, they demonstrated a highly statistically and clinically substantial reduction in dysmenorrhea, deep dyspareunia, nonmenstrual pelvic pain, and dyschezia scores. Lesion dimensions progressively decreased at the 6- and 12-month assessments. The most frequent adverse events were headache and decreased libido.

Two systematic reviews compared DNG with COCs, and another one compared DNG with GnRH analogues.

Piacenti et al. [97] compared the efficacy and tolerability of DNG and COCs by pooling data from four RCTs and one cohort study. No significant difference was observed between the two regimens in pain symptom relief, except for deep dyspareunia, which was reduced to a greater extent in COC users. The incidence of treatment-related adverse events, including unexpected bleeding, nausea, vomiting, headaches, hot flashes, and hair loss, was similar in both groups.

Gu et al. [98] conducted a meta-analysis including eight studies and observed that DNG was superior to COCs in alleviating overall endometriosis-associated pain and in improving HRQoL. However, DNG was found to be inferior to COCs when considering nonmenstrual pelvic pain and dyspareunia specifically. The COC group experienced a greater weight increase, though the incidence of all other adverse events was similar between the DNG and the COC groups.

Dick et al. [99] compared DNG with both GnRH agonists and antagonists. To this end, they conducted a systematic review with a network meta-analysis, including eight studies and a total of 3259 patients. High-dose GnRH antagonists were superior to DNG in reducing nonmenstrual pelvic pain. Regarding dyspareunia relief, leuprorelin was superior to DNG, but DNG was superior to GnRH antagonists. High-dose GnRH antagonists were the most effective at reducing dysmenorrhea. However, DNG had the best safety profile and was the compound with the least frequent and severe adverse effects.

Studies comparing progestins and COCs with GnRH agonists and antagonists may appear obsolete nowadays because GnRH analogues should not be considered an alternative to these therapies (i.e., an “either-or” approach), but rather a step-up in management when first-line medications fail due to ineffectiveness or intolerability. Therefore, direct comparisons seem useful mainly for quantifying the net benefit of GnRH analogues over first-line medications in cost–benefit analyses [100].

Several systematic reviews have assessed the effects of hormonal medications as a postoperative measure aimed at preventing symptom and lesion recurrence. Five of them have focused specifically on the effects of DNG [101,102,103,104,105]. In brief, all of the above overviews consistently concluded that using DNG after conservative surgery for endometriosis dramatically reduces the risk of postoperative symptoms and lesion recurrence, albeit no more effectively than other compounds. Erratic bleeding was the most frequently reported adverse effect. These results are inconsistent with those of Xiong et al. [24], who conducted a network meta-analysis on the effectiveness of postoperative medical treatments after conservative surgery for endometriosis, and stated that, of the eight postoperative adjuvant drugs evaluated, including DNG, “only LNG-IUS […] produced a statistically significant reduction in recurrence. The other drugs showed no significant differences when compared to placebo.”

b. Norethisterone acetate

Norethisterone acetate (or norethindrone acetate) is FDA-approved for treating endometriosis at a dose of 5 mg per day. However, reducing the dose is important because NETA is partly converted to EE. The mean conversion ratio of NETA to EE is between 0.7% and 1%. This translates to a dose of approximately 6 μg of EE per mg of NETA [106]. Thus, 5 mg of NETA would be equivalent to an oral dose of 20–30 μg of EE [107], although, according to Huvinen et al. [108], 5 mg of NETA corresponds to 10 μg of EE. This prevents estrogen-deprivation effects, including a reduction in bone mineral content. However, long-term therapeutic doses of NETA moderately increase the thromboembolic risk [108]. Several clinical studies published in the past 15 years have consistently demonstrated that a 2.5 mg daily dose is sufficient to inhibit ovulation and suppress menstruation, achieving results equivalent to the 5 mg dose. This low dose may also limit the typical androgenic adverse effects of NETA, such as seborrhea, acne, hair loss, and weight gain.

Finally, NETA is one of the least expensive hormonal treatments available for endometriosis. This is important as financial accessibility of treatments is one of the patients’ priorities [109]. In fact, a recent survey revealed that financial strain is a significant concern, emphasizing the importance of affordable care [110].

As no systematic review is available on the effects of NETA at the 2.5 mg daily dose in patients with symptomatic endometriosis, the available studies are here individually synthesized.

In a patient-preference trial, NETA 2.5 mg per day and excisional surgery produced similar reductions in severe deep dyspareunia and a comparable moderate degree of satisfaction at 12-month follow-up [111].

In a before-and-after study, the overall pain relief, psychological status, sexual functioning, and HRQoL were similar in NETA 2.5 mg users and in DNG users. After six months of treatment, approximately 70% of participants in both groups were satisfied. However, 58% of NETA users tolerated the medication well compared with 80% of DNG users [112].

Del Forno et al. conducted a similar retrospective comparison in 135 women with ovarian endometriomas who used DNG (*n* = 69) or NETA (*n* = 66) for 12 months [113]. The reduction in endometrioma size was similar in both groups. A significant between-group difference in the relief of all pain symptoms favored DNG. Furthermore, NETA users reported bleeding/spotting and weight gain more frequently than DNG users did.

Vercellini et al. also assessed the proportion of patients who were satisfied with their treatment after switching from a low-dose COC to NETA due to the COCs’ ineffectiveness in alleviating pain symptoms. After 12 months, 70% of participants were satisfied with NETA treatment. Psychological status, sexual function, and HRQoL improved significantly, despite nearly half of the patients reporting suboptimal drug tolerability [114].

Ferrero et al. treated 82 women with symptomatic rectovaginal endometriosis with NETA, 2.5 mg/day, plus letrozole, 2.5 mg/day, or NETA alone for six months. Users of NETA plus letrozole reported a significantly greater relief from pelvic pain symptoms than users of NETA alone. However, due to the adverse effects associated with letrozole use, the proportion of satisfied participants was 56% in the combined drug group and 64% in the NETA-alone group [115].

An identical comparison was conducted specifically in 40 patients with ovarian endometriomas to evaluate variations in cyst size and pain symptoms. At the 6-month assessment, endometrioma volume decreased in both groups, though with a significant between-group difference in favor of the combined drug regimen. Pain relief and satisfaction with treatment were similar among the 20 patients who used NETA plus letrozole and the 20 patients who used NETA alone. The same research group treated 40 symptomatic women with gastrointestinal symptoms associated with colorectal endometriosis with NETA, 2.5 mg a day [115]. Dysmenorrhea, catamenial diarrhea, and rectal bleeding were completely relieved, and nonmenstrual pelvic pain, deep dyspareunia, dyschezia, intestinal cramping, and passage of mucus improved significantly. However, constipation, abdominal bloating, and a feeling of incomplete evacuation after bowel movements were not alleviated. After study completion, 57% of the patients with irritative-type bowel symptoms requested to continue NETA treatment compared to only 17% of those with subocclusive-type symptoms.

Morotti et al. reported the results of long-term treatment with NETA, 2.5 mg/day, in 103 patients with symptomatic rectovaginal endometriosis. Adverse effects caused withdrawal in 16 (38%) women. Of the 61 (59%) participants who completed the study, 42 (68%) were satisfied with the treatment received, corresponding to 41% (42/103) of those originally enrolled [116].

All pain symptoms were significantly improved during NETA treatment. After five years of NETA treatment, the rectovaginal lesion decreased in 32% of the 59 patients evaluated, remained stable in another 32%, but increased in 12% of cases. Overall, the effectiveness of NETA for treating symptomatic rectovaginal endometriosis seemed suboptimal in this study, with less than half of the patients accepting or tolerating long-term treatment.

Scala et al. conducted a patient-preference study to compare the proportion of satisfied patients after using a COC containing 150 μg of LNG and 30 μg of EE (*n* = 50) with a 91-day extended-cycle, or NETA, 2.5 mg/day (*n* = 50), for 12 months. By the end of the study period, 68% of COC users and 82% of NETA users reported satisfaction with their treatment. Both regimens similarly relieved pain symptoms. The number of days with unexpected bleeding was significantly higher in COC users than in NETA users [117].

c. Intrauterine levonorgestrel

The 52 mg LNG-IUD delivers a mean daily dose of 20 μg of LNG into the uterine cavity. The amount of progestogen released by the system decreases progressively over time, resulting in correspondingly decreasing LNG plasma concentrations. Seeber et al. demonstrated that the mean LNG plasma level after the insertion of the medicated IUD was 191 ± 71 pg/mL in the first year, 157 ± 68 pg/mL in the second year, and 134 ± 41 pg/mL in the third year. Levels remained substantially stable up to the sixth (133 ± 38 pg/mL) and seventh years (133 ± 48 pg/mL), decreasing only during the eighth year (117 ± 45 pg/mL) [118].

These findings confirm that the effect of the LNG-IUD is not limited to the uterus, which explains the mainly androgenic adverse effects frequently reported by users. The risk of depression may also increase [119,120], and libido may decrease [121].

Wang et al. conducted a systematic review and meta-analysis to compare the effect of the 52 mg LNG-IUD with that of different systemic medications in relieving dysmenorrhea associated with endometriosis or adenomyosis [122]. After pooling the results from six RCTs in which a visual analog scale (VAS) was used to measure dysmenorrhea severity in patients with endometriosis, no difference was observed between LNG-IUD users and systemic medication users at 6- and 12-month follow-ups. However, triptorelin performed better at subgroup analysis. The LNG-IUD was associated with a higher risk of irregular bleeding. The HRQoL was similar between LNG-IUD and triptorelin users, but higher in LNG-IUD users than in etonorgestrel implant users. A total of 42 RCTs were considered that included patients with adenomyosis in whom dysmenorrhea severity was measured with a VAS. At the 3-, 6-, 9-, and 12-month evaluations, the reduction in dysmenorrhea scores was similar in the LNG-IUD group and in the systemic medication group. No difference was observed between the groups in blood loss, which was measured semi-quantitatively with a pictorial chart.

Song et al. pooled the results of four RCTs and three cohort studies, including 491 participants who used the 52 mg LNG-IUD as a postoperative preventive measure. The effect of the 52 mg LNG-IUD in reducing pain after surgery was comparable to that of GnRH analogs [123]. The LNG-IUD substantially decreased the lesion recurrence rate, with an effect comparable to that of COCs and danazol. Patient satisfaction was significantly higher with the LNG-IUD than with COCs. However, vaginal bleeding was significantly more frequent in the LNG-IUD group than in the GnRH analog group. Improvements in HRQoL were similar in the LNG-IUD group and the comparison group.

Different conclusions were drawn in the Cochrane systematic review by Gibbons et al. [124] about the effect of postoperative use of the 52 mg LNG-IUD. Four RCTs with a total of 157 patients were considered. The certainty of the evidence was judged to be very low to low primarily due to the serious risk of bias and imprecision. The authors are uncertain whether the LNG-IUD improves dysmenorrhea and HRQoL at 12 months compared to expectant management. A higher rate of satisfaction with LNG-IUD compared to expectant management was observed in two studies, but data pooling was not possible. Melasma and bloating occurred more frequently in LNG-IUD users; however, no differences were observed in other adverse events. The authors are also uncertain whether the LNG-IUD is more effective than GnRH antagonists at improving dysmenorrhea and CPP after 12 months. Irregular bleeding was reported more frequently by LNG-IUD users, whereas vasomotor symptoms were the more frequent complaint in GnRH antagonist users. The authors concluded that there is no high-quality evidence supporting the use of the LNG-IUD postoperatively to reduce endometriosis-related pain or improve operative outcomes.

Finally, it should be noted that the LNG-IUD does not consistently suppress ovulation, except for a few months after insertion. This is relevant because endometriomas generally develop from (hemorrhagic) corpora lutea. Chen et al. conducted an RCT on 80 patients undergoing ovarian endometriotic cyst excision to evaluate whether the LNG-IUD prevents postoperative endometrioma recurrence. At the 30-month follow-up, ovarian endometriomas were detected in 10 of 40 LNG-IUD users (25%) compared to 15 of 40 of the participants who were allocated to postoperative expectant management (37.5%), with no significant between-group difference [125].

d. Drospirenone

Drospirenone is a synthetic progestin structurally related to spironolactone. It has anti-mineralcorticoid, anti-androgenic, and anti-gonadotropic properties, and no estrogenic activity. It decreases salt and water retention and may lower blood pressure. The anti-androgenic potency of DRSP is about one-third of that of cyproterone acetate. The half-life of DRSP is approximately 30 h [126]. Limited data is available regarding its use in patients with endometriosis.

Shim et al. [127] retrospectively analyzed the effect of 4 mg/day of DRSP used continuously (i.e., skipping the four placebo pills) in 61 symptomatic adolescents, 59 of whom had laparoscopically confirmed endometriosis. Most of the patients had superficial peritoneal implants, had previously tried NETA, and had contraindications to estrogen use. After a median follow-up period of 162 days, dysmenorrhea improved in 62% of participants, and pelvic pain improved in 54%. However, 23% of patients discontinued treatment due to adverse effects. The most common reason for discontinuation was unpredictable bleeding, experienced by 44% of participants.

Garbo et al. [128] prescribed DRSP, 4 mg/day continuously to 136 adolescents with dysmenorrhea (80/136) and endometriosis (61/136). The aim was menstrual suppression. After a median treatment period of approximately one year, 44.3% of participants experienced breakthrough bleeding, which was the main reason for discontinuing the drug. Nevertheless, 44/52 patients with dysmenorrhea and 28/36 of those with pelvic pain reported symptomatic improvement.

2. Oral contraceptives containing body-identical estrogens: combining limited endometriosis stimulation with reduced thromboembolic risk

In general, COCs containing EE have been shown to cause an increase in procoagulant factors (i.e., factor II, factor VIII, factor IX, and fibrinogen). Additionally, natural anticoagulant activity is reduced (antithrombin, protein C, and protein S). Moreover, an increase in tissue-plasminogen activator, plasmin-alpha2-antiplasmin complexes, and D-dimer has been observed. These imbalances between pro- and anti-coagulation mechanisms and fibrinolysis are similar to those observed during pregnancy [129]. The consequent rise in VTE risk is directly proportional to both the EE dose and to the type of progestogen used [25,130].

Progestogens that possess androgenic properties have been shown to counteract the pro-thrombotic effect of EE. Conversely, neutral or even anti-androgenic progestogens exhibit a limited or no effect [130,131]. This explains the disparity in VTE risk associated with second-generation versus third- and fourth-generation EE-containing COCs [85]. Both E2 and estetrol (E4), a native estrogen synthesized by the fetal liver during pregnancy, have a reduced effect on coagulation and fibrinolysis when compared to EE. The modest increase in the hepatic synthesis of procoagulant factors results in a VTE risk that is comparable to or lower than that associated with a second-generation COC, such as the standard reference combination of EE 20 μg/LNG 100 μg [25,26,27,131,132,133]. Considerations analogous to those concerning VTE also apply to the rarer arterial thrombotic events (i.e., stroke and myocardial infarction) [134,135].

The interesting concept of the gradient of “total estrogenicity” of individual hormonal combinations used in contraception and hormone therapy is presented graphically in Douxfils et al. [136].

With respect to the assessment of VTE risk, Meiadi et al. [34] have categorized available hormonal contraceptives into high-, medium-, and low/no-risk groups. Birth-control pills containing high-dose EE, as well as those with 20–30 µg EE combined with desogestrel, gestodene, DRSP, and cyproterone acetate, were included in the high-risk group, together with the estrogen-progestogen patch and the vaginal ring. All other COCs and DMPA were classified as medium-risk contraceptives. Exclusively POPs, progestogen implants, and LNG-IUDs were considered to be at low or no risk.

The availability of estrogen-progestogen combinations containing body-identical estrogens is of particular interest in the long-term treatment of endometriosis. In fact, their limited estrogenic potency means that the stimulation of endometriotic lesions is limited, and the VTE risk is reduced.

As reported by Kim et al. [137], the oral administration of 1 mg of E2 hemihydrate to postmenopausal women resulted in a mean serum E2 concentration of 64.6 pg/mL. This corresponds to the mean concentration observed when administering conjugated equine estrogens at the 0.45 mg dose (60.1 pg/mL). The results were similar when 1 mg of E2 valerate (E2V) was used. Several investigators emphasized the equivalence of 5 μg EE to approximately 1 mg micronized E2. Thus, low-dose COCs containing 20 μg EE would have an estrogenic potency that is equivalent to 4–6 times the physiological dose of E2 [89,138,139]. Consequently, the use of EE-containing COCs, even at low doses, implies a needlessly increased risk of VTE compared with estrogen-progestogen combinations containing body-identical estrogens [27]. At the same time, the risk of endometriotic lesion stimulation is potentially increased. Moreover, supraphysiologic estrogen concentrations may exacerbate neuroinflammation through microglial activation [140]. This detrimental effect could further disrupt interoceptive processing pathways, heightening sensitivity to sensory stimuli in women with central sensitization syndrome. On this basis, we do not recommend the use of EE, except when a patient is already on an EE-containing COC with evidence of substantial pain relief, optimal tolerance, and favorable physical and US findings. Accordingly, here we will synthesize exclusively the results published on monophasic estrogen-progestogen combinations containing E2, E2V, and E4.

a. Estradiol hemihydrate plus nomegestrol acetate

This COC is of particular interest for patients with endometriosis because it combines a low dose of body-identical E2 with nomegestrol acetate (NOMAC), a progestogen with a notably prolonged half-life. Nomegestrol acetate exerts a marked anti-estrogenic activity and atrophic effect on the endometrium, and is associated with a limited likelihood of withdrawal bleeding [141]. We identified four studies (three cohort studies and one RCT) that focused on the effect of a combination of E2 hemihydrate, 1.5 mg, and NOMAC, 2.5 mg, in patients with symptomatic endometriosis.

Caruso et al. [142] conducted a prospective, patient-preference, cohort study on 162 symptomatic women with a clinical diagnosis of endometriosis, 99 of whom chose E2-NOMAC used cyclically with a 24/4 day regimen for six months, whereas 63 participants declined the use of hormonal treatment and used common NSAIDs. Women with a US diagnosis of adenomyosis, ovarian endometriomas, and rectovaginal endometriosis were excluded. Significant between-group differences were observed at 3- and 6-month evaluations in all pain symptoms, sexual functioning, and HRQoL. A total of 68 (88%) participants using the COC were satisfied with their treatment, 5 (7%) were somewhat satisfied, and 4 (5%) were neither satisfied nor dissatisfied. In the NSAIDs group, 27 participants (69%) were neither satisfied nor dissatisfied, 8 (21%) were dissatisfied, and 4 (10%) were very dissatisfied.

Mariani et al. [143] retrospectively reviewed data from 39 patients with ovarian endometriomas and deep infiltrating endometriosis (DIE) who used E2/NOMAC cyclically or continuously based on their preference. After six months of treatment, a significant reduction in VAS pain scores and endometrioma diameter was observed, whereas DIE lesion dimensions remained unchanged.

A further cohort study evaluating the effect of various COCs, including E2/NOMAC, is outlined in the subsection on the E4/DRSP combination [144].

The only RCT available on the effect of E2/NOMAC on endometriosis-associated pain was conducted by Caruso et al. [138], who compared this COC used cyclically (*n* = 99) with DNG, 2 mg daily used continuously (*n* = 98). Once more, patients with a clinical and US diagnosis of adenomyosis, ovarian endometriomas, and DIE were excluded from the study. At the 12-month assessment, dysmenorrhea, deep dyspareunia, and CPP VAS scores showed substantial improvements compared to the baseline values, with no significant between-group differences. However, participants allocated to DNG reported better sexual function and HRQoL compared with those allocated to E2/NOMAC. Approximately one woman out of 10 discontinued the allocated treatment due to irregular bleeding. Overall, only one-third of women using E2/NOMAC cyclically experienced amenorrhea, compared with two-thirds of those using DNG.

b. Estetrol plus drospirenone

The combination of E4, 15 mg, and DRSP, 3 mg, seems to be of benefit for headache, fatigue, water retention, weight gain, swelling, breast tenderness, as well as anxiety, mood swings, and irritability [145]. The E4/DRSP combination shows reduced estrogenic activity compared to COCs containing EE. This impacts coagulation and surrogate markers for VTE risk. Kobayashi et al. [146] conducted a phase II, multicenter, RCT to evaluate the effects of E4/DRSP on coagulation and fibrinolysis in patients with endometriosis. A total of 88 participants received either E4/DRSP 3 mg or EE 20 µg/DRSP 3 mg for 12 weeks. The E4/DRSP group exhibited a substantially lower effect on coagulation and fibrinolysis compared to the EE/DRSP group. Specifically, the activated protein C sensitivity ratio was increased by approximately 4-fold in the EE/DRSP group compared to the E4/DRSP group, and the D-dimer levels were 4.7-fold higher in the EE/DRSP group than in the E4/DRSP group.

However, metrorrhagia was reported as the most common treatment-related adverse event [147], making this combination a possible second choice, selectively in COC users who experience estrogen-induced water retention and negative affect. Three studies are available on the use of the E4/DRSP combination used cyclically to relieve pain in patients with symptomatic endometriosis: two RCTs and one cohort study.

Harada et al. [139] conducted a multicenter, placebo-controlled, double-blind RCT on 162 Japanese women. At the six-month assessment, a −8.5 mm greater reduction in mean VAS score was observed in the E4/DRSP arm compared to the placebo arm. A higher proportion of participants in the E4-DRSP group reported a VAS score reduction ≥ 50% from baseline values (53.2% vs. 29.6%, respectively). No significant between-group differences were observed in dyspareunia and dyschezia VAS scores. Intermenstrual bleeding was the most common treatment-emergent adverse event among E4/DRSP users, occurring in 51.9% of participants during the first cycle and still in 24.4% of them after six cycles.

The second, multicenter, active-controlled, open-label RCT was conducted by Harada et al. [148] on 88 Japanese patients with symptomatic endometriosis. The study is noteworthy also because the active comparator contained the same progestin at the same dose (DRSP, 3 mg), and the commonly used estrogen at a low dose, i.e., EE, 20 μg. Therefore, the only differences between the two treatments were the type of estrogen and the modality of use (cyclically for E4/DRSP and continuously for EE/DRSP). The participants received the assigned treatment for 12 weeks. The nonmenstrual VAS score reduction over time, the proportion of responders, and the proportion of satisfied participants, as indicated by the Patient Global Impression of Improvement, were all significantly higher in the E4/DRSP arm than in the EE/DRSP arm. These findings support the hypothesis that even low doses of EE do not allow full suppression of endometriotic lesions. Intermenstrual bleeding was the most frequent adverse event observed in both the E4/DRSP group (68.9%) and the EE/DRSP group (73.2%), confirming that DRSP generally provides suboptimal bleeding control. Its use should be reserved for patients who experience bloating, weight gain, acne, depressed mood, and reduced libido with other progestins.

Caruso et al. [144] conducted a cohort study to compare the effects of five COCs used cyclically [four monophasic COCs, i.e., EE 30 μg/DNG 2 mg (*n* = 36); EE 20 μg/DRSP 3 mg (*n* = 40); E2 1.5 mg/NOMAC 2.5 mg (*n* = 38); E4 15 mg/DRSP 3 mg (*n* = 34); and one multiphasic COC, i.e., E2V/DNG (*n* = 40)] and DNG 2 mg daily used continuously in patients with endometriosis-associated dysmenorrhea, dyspareunia, and CPP. Women with US-diagnosed ovarian endometriosis and DIE were excluded. At the 6-month assessment, participants using E2/NOMAC, E2/DNG, or E4/DRSP showed a greater improvement in CCP than those using the COCs containing EE. Patients using E4/DRSP demonstrated a comparable pain improvement to those using DNG, and a greater improvement than patients using COCs containing E2/NOMAC. Patients using E2 and E4-containing COCs, and DNG, experienced a greater improvement in dysmenorrhea and dyspareunia than those using EE-containing COCs. The authors concluded that, for patients with symptomatic endometriosis-associated pain, COCs containing E2 or E4 could constitute a more effective treatment than COCs containing EE. Unfortunately, the study’s suboptimal design, the selection criteria, and the unclear modality for treatment assignment greatly limit the generalizability of these findings.

c. Estradiol valerate plus dienogest

In Europe, an oral combination is available containing E2 valerate (E2V), 1 mg, and DNG, 2 mg. Estradiol valerate is an esterified form of natural E2 that acts as a prodrug. Following oral administration, E2V is hydrolyzed to E2 in the gastrointestinal tract and liver. One mg of E2V is equivalent to 0.76 mg of E2 [137,149]. While this estrogen-progestogen combination is approved for menopause hormone therapy, it seems to be a promising off-label alternative to be included among the first-line hormonal medications category. In fact, the serum E2 levels observed in postmenopausal women using E2V, 1 mg/day, are between 65 and 75 pg/mL, corresponding to the levels observed when using conjugated equine estrogens, 0.45 mg/day [137]. Given that a serum E2 level of 60 pg/mL is necessary to prevent osteoporosis [150,151], this combination appears to be ideal for long-term suppression of endometriosis. The rationale behind this is that a low dose of a biosimilar estrogen is added to the standard dose of the progestogen that has been most studied for the medical treatment of endometriosis. This could allow for the long-term use of DNG, while potentially preventing the negative effects of hypo-estrogenism, including bone mass loss, with limited thromboembolic risk. Regrettably, there are no available reports on the effect of this combination in patients with symptomatic endometriosis. Therefore, studies are warranted to assess the purported advantages of adding E2V, 1 mg, to the usual dose of DNG.

## 4. Second-Line Drugs for Nociceptive Pain

GnRH analogues, i.e., GnRH agonists and antagonists, are the second-line hormonal medications for symptomatic endometriosis [38,39]. As the group denomination itself indicates, GnRH analogues are not an alternative to progestogens and estrogen-progestogen combinations. They should be used when there is no hormonal alternative because first-line drugs have failed. In practice, this means that approximately two-thirds of patients experiencing mainly nociceptive pain will never require a GnRH analogue. However, for the remaining third, these medications may constitute a very effective therapeutic option that can induce substantial pain relief and markedly improve health-related QoL [152,153]. Depot agonists and full-dose antagonists are particularly indicated when complete amenorrhea is not achieved with progestogens and estrogen-progestogens, as irregular, unexpected bleeding can cause pain, anxiety, and dissatisfaction, and reduce treatment adherence. GnRH analogues constitute the most effective hormonal option currently available to induce amenorrhea, minimize the risk of breakthrough bleeding and spotting, and relieve endometriosis-associated pain [154]. Patients must never face continued suffering due to healthcare providers denying them access to this pharmacologic alternative.

A detailed description of individual components with relevant pharmacokinetic and pharmacodynamic properties will not be provided here. Indeed, while various progestogens and estrogen progestogen combinations possess distinct characteristics, including varying efficacy, safety, and tolerability profiles, as well as bleeding control, all monthly or three-monthly depot GnRH agonists and full-dose, daily oral GnRH antagonists ultimately achieve the same outcome, i.e., a complete ovarian function suppression and a profound hypoestrogenic state, albeit through different mechanisms [153]. Both drug classes share similar efficacy and tolerability profiles as well as bleeding control capabilities [153,155]. These characteristics are directly proportional to the degree of hypoestrogenism achieved [100,156,157]. Safety aspects, including bone mineral density (BMD) variation, are also associated with the lack of circulating estrogens, and not with intrinsic features of the different compounds. In summary, for an individual patient with symptomatic endometriosis, there are minimal changes, if any, in treatment outcomes when using a depot GnRH agonist or a high-dose oral GnRH antagonist.

The purported benefits of GnRH antagonists over GnRH agonists seem questionable (see Othman et al. [153], for detailed considerations on potential differences), except for the possibility of immediately discontinuing the drug in oral GnRH antagonist users. However, cases of intolerable side effects so severe that they require the immediate interruption of the medication are infrequent. The flare-up phase, which is typical of GnRH agonists, but not of GnRH antagonists, can be effectively prevented by administering the initial injection during the midluteal phase or at the commencement of a 10-day course of any progestogen or estrogen-progestogen combination [155]. The preference for oral over intramuscular administration seems to be a matter of personal choice, with some patients opting for the convenience of tablets and others concerned about the daily reminder and potential for forgetfulness. The need for healthcare providers to administer GnRH agonist injections can be substantially reduced by opting for three-monthly depot formulations instead of monthly depot formulations. Moreover, the three-monthly 11.25 mg triptorelin depot can be safely administered every four months instead of three months, ensuring sustained ovarian suppression without loss of efficacy [158], thereby reducing the need for healthcare assistance and lowering costs [159].

As outlined below, we believe that combining a GnRH antagonist with add-back therapy in a single tablet could enhance compliance, but it hinders the ability to tailor the hormonal environment to meet individual needs and preferences.

Finally, we are reluctant to consider the use of a GnRH antagonist at low doses (i.e., elagolix 150 mg daily; linzagolix 100 mg daily) as a reasonable alternative when a shift from a first-line to a second-line medication is required. This change is typically driven by insufficient efficacy and/or tolerability, including inadequate control of erratic bleeding. In patients experiencing medical failure with progestogens and COCs, it is important to consider the most effective alternative treatment to improve their HRQoL promptly. However, low-dose GnRH antagonists have been shown to have a lower likelihood of amenorrhea and pain relief compared to full-dose GnRH antagonists [155]. In addition, since GnRH antagonists at low doses do not systematically suppress ovulation, it is not possible to determine whether the amenorrhea is a drug-induced effect or due to an unrecognized ongoing pregnancy. This event occurred in nearly 1% of participants in phase-3 trials [160], and the percentage could be higher when low doses are used in routine settings instead of research settings. This would necessitate performing repeat pregnancy tests at regular intervals. Lastly, GnRH antagonists at low doses are no less expensive than high-dose formulations.

A Cochrane systematic review is available on the effects of GnRH analogues compared with a placebo or other hormonal treatment options in patients with endometriosis [161]. A total of 72 RCTs comprising 7355 participants were included in the study. The quality of the evidence was judged to be very low to low. After three months of therapy, GnRH analogues may decrease pelvic pain, dysmenorrhea, and dyspareunia compared with placebo (low-certainty evidence). After six months of therapy, GnRH analogues may reduce pelvic pain to a slightly greater extent than danazol (very low-certainty evidence). No trial at low risk of bias was identified comparing GnRH analogues versus LNG-IUD. The authors concluded that there may be a minor difference in favor of GnRH analogues compared to placebo or oral or injectable progestogens in overall pelvic pain relief. However, they are uncertain regarding potential differences when comparing GnRH analogues with danazol, intra-uterine progestogens, or gestrinone. Due to the very low certainty of the evidence, they recommend interpreting the results with caution.

To the best of our knowledge, Yan et al. [156] were the first investigators to conduct a systematic review and network meta-analysis on the effect and safety of GnRH antagonists for the treatment of moderate to severe endometriosis-associated pain. The authors included six RCTs comprising a total of 2796 participants and 10 different drug doses. As expected, the majority of outcomes related to efficacy and safety exhibited a dose–response relationship. The best treatments for pain relief were found to be elagolix 400 mg, linzagolix 75 mg, and linzagolix 200 mg, while the best treatments for the relief of dysmenorrhea were relugolix 40 mg, elagolix 400 mg, and relugolix 20 mg. Only high-dose treatments were significantly associated with most outcomes related to HRQoL. A significantly increased incidence of adverse events was observed in users of relugolix 40 mg and elagolix 400 mg.

Xin et al. [157] conducted a second systematic review and network meta-analysis to assess the effect of different GnRH antagonists in patients with moderate-to-severe endometriosis-associated pain. Elagolix 400 mg daily was the most effective compound in reducing pelvic pain, dysmenorrhea, and dyspareunia, whereas relugolix 40 mg daily was the best GnRH antagonist in reducing analgesic use. The proportions of participants experiencing treatment-related adverse effects and discontinuation because of them were highest in the relugolix 40 mg and elagolix 250 mg groups, whereas rates of hot flush and headache were highest in relugolix 40 mg and elagolix 150 mg users. The authors confirmed that a dose–response relationship exists for both efficacy and tolerability of oral GnRH antagonists.

Subsequent systematic reviews have confirmed the equivalence among different GnRH antagonists and the substantial reduction in adverse effects, including the impact on bone density, when used in combination with an add-back therapy [100,162,163].

Open-label extension studies have compellingly demonstrated that GnRH antagonists retain their efficacy when used for prolonged periods and can be safely used for years, provided they are combined with adequate add-back therapy [164,165,166].

## 5. Add-Back Therapy: From Disregarded Adjunct to Pivot of Customized Medical Treatment

When considering the use of GnRH analogues, the primary debate typically centers on the differences between agonists and antagonists, as well as the potential advantages or disadvantages of different dosages. Over time, the recommendation to combine analogues with any “add-back therapy” when extending treatments beyond a few months gradually became a sort of generic caveat, implying that the specific type of hormones used is of lesser importance, as long as vasomotor symptoms are relieved, and bone mineral content is preserved [155].

We propose an alternative perspective: the drug used to suppress ovarian function is substantially unimportant, provided a steady and deeply hypoestrogenic milieu is induced. This is the mechanism through which endometriosis is controlled by GnRH analogues. Upon this hormonal ground, which is identical and is associated with similar adverse effects regardless of the GnRH analog used, add-back therapies can be customized to align with individual patient profiles and preferences. For instance, progestogens with androgenic properties, such as NETA, should be avoided in women with a tendency to develop acne and seborrhea. The use of a progestogen structurally related to spironolactone, such as DRSP, could be indicated for women particularly concerned about water retention and weight gain.

Moreover, estrogens should not necessarily be combined, nor administered exclusively by the oral route. As the liver’s first-pass metabolism of estrogens influences thrombotic risk, the transdermal applications of gels containing body-identical E2 can be an effective strategy to limit the hepatic synthesis of pro-thrombotic factors [167]. In addition, the administration of oral or vaginal micronized progesterone, 200 a day, could mitigate the increase in risk of breast cancer [168,169,170]. Dydrogesterone, an isomer of natural progesterone, can also be used at an oral dose of 10 g/day. Alternatively, there are estrogen-progestogen transdermal patches available that generally provide fairly predictable and stable serum hormone levels [171]. In patients experiencing troublesome bleeding with micronized progesterone or dydrogesterone, body-identical E2 can be used transdermally in combination with a progestogen such as DNG, taken orally [155].

Finally, oral tibolone, 2.5 mg a day, is a popular, effective, and inexpensive add-back therapy that does not stimulate endometriotic lesions due to its androgenic properties [155].

The selection of the type of estrogen, the type of progestogen, the doses of the two hormones, and the respective route of administration allows for the customization of add-back therapy based on individual needs, preferences, and tolerability, while improving safety. In the end, the effects experienced by women when GnRH analogues are combined with add-back therapies are attributable to the latter medications, rather than the former ones. Add-back therapies are not intended to manage endometriosis beyond the action of GnRH analogues.

When using oral GnRH antagonists, the above considerations obviously apply to high drug doses not combined with an estrogen-progestogen in the same tablet [100]. In this regard, maintaining the GnRH antagonist and the add-back therapy as separate treatments offers the additional advantage of temporarily discontinuing only the latter one for a few days in case of prolonged spotting or breakthrough bleeding while continuing the administration of the antagonist.

Regarding the absorption of body-identical E2, a daily dosage of 0.5 mg administered as a gel is approximately equivalent to 0.25 μg delivered daily by a matrix-type patch [172], resulting in a mean serum E2 level of approximately 60 pg/mL [173]. However, when using body-identical E2 transdermally, it may be advisable to periodically measure the E2 serum level, as absorption may vary and be influenced by various factors, including the site of application [171]. The goal is to maintain serum E2 levels around 60 pg/mL to prevent bone resorption [150] without stimulating endometriotic lesions. If a transdermal gel is used, its daily amount can be adjusted based on E2 serum levels to ensure adequate bone protection [174]. Similarly, serum progesterone levels should be measured when using oral or vaginal micronized progesterone 200 mg capsules, as absorption and serum concentrations are also highly variable [175].

While the maximum duration of use of GnRH analogues combined with add-back therapy is currently limited to two years in most countries, some patients request or require prolonged therapy beyond this limit. In such cases, it is advisable to perform periodic bone mineral density measurements to monitor for any potential decreases that may require an increase in E2 dosage or a transition to a progestogen with androgenic properties.

## 6. Therapeutics for Neuropathic and Nociplastic Pain in Patients with Endometriosis-Associated “High Sensitivity to Pain-Inducing Stimuli” [176]

Patients with endometriosis frequently experience different types of pain, i.e., (i) peripheral nociceptive pain caused by tissue injury induced by inflammatory mediators released at ectopic endometrium sites; (ii) neuropathic pain caused by injury (compression or infiltration) to the somatosensory neurons; and (iii) central nociplastic pain, which is characterized by amplification of ascending pain signals, decreased descending pain input inhibition, and generalized sensory sensitivity [29]. Central sensitization appears to be caused by repetitive incoming painful stimuli, followed by altered pain processing, a high reactivity state characterized by increased evoked activity of nociceptive neurons, a lowered pain threshold, and the involvement of additional structures via viscero-visceral cross-sensitization, which affects organs that share the same nerve segmentation. These changes may result in hyperalgesia (a pain sensation that is disproportionate to a noxious stimulus) and allodynia (a pain sensation that arises from a non-noxious stimulus). Additionally, central sensitization shifts the condition from local to systemic, requiring management as a chronic illness [40].

From a conceptual standpoint, nociceptive and nociplastic pain coexist. Therefore, it is recognized that many patients experience pain states in which more than one type of pain is present. For this reason, the different types of pain should not be considered as exclusive categorical labels, but rather, pragmatically, as possible concurrent and/or contributing mechanisms to the patient’s pain. According to Vincent and Horne [31], “up to 40% of patients with symptomatic endometriosis have a neuropathic-like component and a similar proportion have features consistent with a nociplastic mechanism (e.g., widespread pain, fatigue and ‘brain fog’)”.

This review does not address the mechanisms that lead to a transition from peripheral nociception to central sensitization/nociplastic pain in detail, nor does it define the methods for diagnosing non-nociceptive pain components. For more information, the reader can refer to the following sources: the American College of Obstetricians and Gynecologists Practice Bulletin, Number 218 on Chronic Pelvic Pain [177], the Society of Obstetricians and Gynaecologists of Canada Guideline No. 445: Management of Chronic Pelvic Pain [40], the Royal College of Obstetricians and Gynaecologists Green-Top Guideline No. 41—The Initial Management of Chronic Pelvic Pain [178], and the best available comprehensive reviews, including those by Lamvu et al. [179], Till et al. [37], Meisenheimer and Carnevale [180], and As-Sanie et al. [29].

When endometriosis-associated pain symptoms are refractory to standard hormonal treatments, non-response may be associated with the development of peripheral and central sensitization [30,31]. Therefore, gynecologists providing care to patients with endometriosis-associated mixed-type pain should try to reduce peripheral nociceptive stimuli and understand how to detect and treat neuropathic and nociplastic pain. These latter components, along with classic nociceptive pain, shape the individual pain phenotype. The therapeutics indicated below are the most frequently used compounds for neuropatic or nociplastic pain. However, the list is not exhaustive, and other drugs not described here could be co-administered or used as an alternative. Moreover, these drugs should be considered as options for managing the nociplastic pain component that persists after hormonal therapy is fully optimized (i.e., after GnRH analogues with add-back therapy do not substantially relieve chronic pelvic pain symptoms).

There is practically no evidence on the efficacy of neuromodulators for endometriosis-associated pain symptoms [31], and limited data are available even on the use of these drugs in patients with CPP of various etiologies or with no obvious pathology. Therefore, the following cautious advice is mainly based on the use of neuromodulators in patients with chronic pain in general. Neuromodulators may be indicated when neuropathic pain is suspected, when overlapping chronic pain conditions are diagnosed, or when myofascial dysfunctions are detected. However, they are generally not indicated when the main or only determinant of the reported symptoms is the typical nociceptive pain caused by endometriosis. Regarding nociplastic pain, we are unaware of studies demonstrating that the available medications described below objectively impact the mechanisms associated with central sensitization, and lead to an overall “reset” of central pain perception in women with symptomatic endometriosis.

Amitriptyline, a tricyclic antidepressant, can be administered at an initial dosage of 10–25 mg/day at bedtime, which can be titrated slowly to a maximum dosage of 75/day at bedtime. Adverse effects, including sedation, dry mouth, constipation, and weight gain, limit treatment adherence [179,180,181,182,183].

Duloxetine, an antidepressant that blocks the reuptake of both serotonin and norepinephrine, is used at an initial dose of 30 mg/day, which can be increased to a maximum of 60 mg/day if there is no response after eight weeks. Adverse effects include sedation, loss of libido, weight gain, dizziness, and headache. Caution is needed when combining duloxetine with other serotonergic medications, such as gabapentinoids and cyclobenzaprine, due to the risk of triggering the dangerous serotonin syndrome [179,180,181,182,183].

Both gabapentin and pregabalin are centrally acting calcium channel blockers. Gabapentin was originally developed as an antiepileptic therapy. Gabapentinoids inhibit the release of glutamate, norepinephrine, and substance P, which are excitatory neurotransmitters that mediate pain transmission and influence central sensitization. The initial gabapentin dose is 100 mg/day at bedtime, which can be titrated by 100 mg per week up to 300 mg/day at bedtime. Any further increase in dosage should be divided into two or three doses. Gabapentin may cause sedation, dizziness, drowsiness, blurred vision, and headaches [179,180,181,182,183]. Gabapentin and amitriptyline are often administered together, but the supposed benefits of this combination are currently undefined [181,182].

The initial pregabalin dose is 25 mg at bedtime and can be increased by 25 mg/day in two or three divided doses up to a maximum suggested dose of 300 mg/day, though the usual dosage for CPP is 75 mg twice per day [40]. Pregabalin may induce somnolence, dizziness, asthenia, difficulty concentrating or thinking, and weight gain [183]. There is no robust evidence demonstrating the superiority of pregabalin over gabapentin in treating neuropathic CPP [182]. Horne et al. [184] conducted a multicenter, placebo-controlled, double-blind randomized trial investigating the effect of gabapentin in women with CPP and no apparent pelvic pathologies identified at laparoscopy. Gabapentin was not superior to placebo in reducing pelvic pain scores, but it was associated with a significantly higher incidence of serious adverse events.

Cyclobenzaprine, a centrally acting skeletal muscle relaxant, is pharmacologically related to tricyclic antidepressants. It is approved for short-term use to relieve muscle spasms in acute conditions, but data on long-term use are limited. In particular, high-quality data on the effect of cyclobenzaprine and other frequently prescribed muscle relaxants in treating CPP are lacking [179]. When pelvic floor dysfunction/hypertonia coexists with endometriosis, a 10 mg dose of cyclobenzaprine can be administered at bedtime, considering its sedating effects may improve sleep dysfunction related to pain [29,37]. Daytime use is discouraged due to the frequently reported drowsiness. Cyclobenzaprine is a serotonergic drug; therefore, co-administration with amitriptyline or duloxetine should be carefully considered due to the risk of triggering a serotonergic syndrome.

In general, amitriptyline and serotonin–norepinephrine reuptake inhibitors may be chosen in women with depressed mood in addition to pain. Gabapentinoids, particularly pregabalin, may be of benefit when anxiety is a complaint. Muscle relaxants, such as cyclobenzaprine, are indicated for patients with myofascial pain, including pelvic floor dysfunction, coexisting with endometriosis [179,180,181,182,183].

Overall, the magnitude of the effect of neuromodulatory medications specifically in patients with endometriosis and mixed types of pain is substantially unknown and derived indirectly from studies on populations with chronic pain in general, and is presumably of similarly limited benefit in reducing pain severity. Physicians must have adequate knowledge of the mechanisms of action, dosages, interactions, adverse effects, and processes for gradually increasing and de-escalating doses of these drugs before prescribing them [185]. In particular, gynecologists lacking specific experience should not co-administer these compounds. Moreover, given the very limited evidence regarding their impact on central sensitization mechanisms in patients with endometriosis, these drugs should mainly be used to relieve neuropathic (peripheral) pain, even though the balance of potential benefits and harms is currently unclear. In addition, combining hormonal and neuromodulatory drugs may reduce adherence because young patients may be less prone than older ones to tolerate polypharmacy.

The use of neuromodulatory drugs should be based on a diagnosis, i.e., identification of the types of pain involved. However, when discriminating between nociceptive and peripheral and/or central sensitization is challenging, or when co-administering different neuromodulators or substantially increasing the dosage is considered, the inexperienced gynecologist should refer the patient to a pain specialist or a pain clinic. Finally, these medications should be used as part of a holistic approach that includes non-pharmacological measures, such as physiotherapy, cognitive behavioral therapy, mindfulness, and lifestyle changes, including adopting a low-FODMAP diet and exercising regularly. This approach should also address coexisting chronic overlapping pain conditions [29,37,177,179].

## 7. Experience-Based Take-Home Messages

Based on the above information and considerations, the following priorities regarding the medical management of endometriosis-associated pain are recapitulated.

1. Medical treatment is not for all

Several conditions preclude the choice of hormonal medications as a treatment option. These include patients with obstructive uropathy, bowel endometriosis with subocclusive symptoms, ovarian cysts with an unclear US appearance, endometriomas larger than 5 cm, and those that develop or grow during suppressive therapy, or in women over 45 years of age. In the latter conditions, malignancy must be ruled out [186]. Patients who refuse hormonal medications and those seeking conception are also excluded from this treatment option.

2. Safety first

“*Hormonal contraceptives are mostly used by otherwise healthy women for non-medical indications. But this does not take away the fact that they are pharmaceutical formulations and with that comes a responsibility when prescribing them. As physicians, we have a duty to ensure that each woman is prescribed the safest hormonal contraceptive for her*”.

(Meaidi [187])

Prolonged therapy is standard for human chronic disorders, and endometriosis is no exception. Therefore, the potential long-term consequences of medications should be carefully pondered, also bearing in mind that progestogens and COCs are used here to treat a potentially disabling condition, not “merely” to provide contraception. This influences the therapeutic balance between potential benefits and harms.

In addition to the associations with venous and arterial thrombotic events [188], risks of meningiomas and cancers should also be considered.

Some population-based studies have demonstrated an increased risk of intracranial meningiomas, often located in the anterior and middle parts of the skull base, when using several progestogens, including cyproterone acetate, NOMAC, chlormadinone acetate, desogestrel, medrogestone, medroxyprogesterone acetate, and promegestone [189]. The effect appears to be cumulative; even low doses may increase the risk when taken over a long period of time [190,191,192]. Data on levonorgestrel are inconsistent. An increased risk has also been reported for this progestogen, based on an analysis of the U.S. Food and Drug Administration Adverse Event Reporting System [193].

However, except for cyproterone acetate, the number needed to harm (NNH) is generally high. For instance, a population-based cohort study using TriNetX data, a U.S. national database of 68 healthcare organizations, found that the relative risk of meningioma was 2.43 (95% CI, 1.77–3.33) among DMPA users and 1.18 (95% CI, 1.10–1.27) among oral MPA users. The NNH was 1152 users for DMPA and 3020 users for oral MPA [194]. In a large, nationwide, Swedish, registry-based study, women who were prescribed DMPA injections had fivefold odds of developing meningioma. However, this corresponds to only two additional cases of meningioma per 10,000 women exposed to DMPA per year [192]. The NNH for one additional intracranial meningioma requiring surgery with desogestrel use has been estimated to be 67,300 women. This risk was no longer detected one year after discontinuing desogestrel [191]. Based on meningioma incidence rates retrieved from the U.S. Surveillance, Epidemiology, and End Results (SEER) Program database (available at: www.seer.cancer.gov), and the cumulative risk in exposed women as well as the attributable cumulative risk increase per 100,000 persons indicated by the European Medicines Agency, the NNH for NOMAC, when used continuously for five years at a daily dose of 2.5 mg by 20- to 34-year-old women, has been estimated to be approximately 3900 [195].

Moreover, since the primary risk factor is age, and patients using progestogens for endometriosis tend to be young, the overall clinical risk seems limited. European regulatory authorities required this risk to be included in the patient information leaflet, but did not issue a recall of these drugs from the market [155]. Women taking progestogens should be monitored for the onset of CNS symptoms, and referral to a neurological service is advised in case of doubt. Most meningiomas that develop during progestogen use regress after discontinuing the drug without the need for demanding surgery [196,197].

Hormonal contraception is associated with a modest increase in breast cancer risk. However, considering the low incidence rate of this malignancy in young women, the absolute risk increase is very limited. Furthermore, this modest increase disappears a few years after discontinuing progestogens or COCs. Conversely, prolonged COC use substantially reduces the risk of ovarian and endometrial cancers and, to a lesser extent, also the risk of colorectal cancer. This protective effect lasts for many years after discontinuing the drugs. Therefore, patients should be reassured that the overall “oncologic balance” is favorable [198].

3. Types of symptoms and lesions

“*Clinicians need to relinquish their role as the single, paternalistic authority and train to become more effective coaches or partners—learning, in other words, how to ask, “What matters to you?” as well as “What is the matter?”*”

(Barry and Edgman-Levitan [199])

We treat people, not “clinical cases”, and each person has specific needs and priorities, regardless of the form, extent, or staging of their endometriosis. Therefore, therapeutic efforts should first focus on the most distressing symptom for each patient. Admittedly, we have mainly addressed hormonal therapies here, which may be costly and associated with adverse effects. We have not considered complementary therapies, such as botulinum toxin application, which may improve outcomes for patients presenting with specific clinical conditions [35,36].

Dysmenorrhea, especially when associated with superficial lesions or endometriomas ≤ 5 cm, can be alleviated by suppressing menstruation with COCs used continuously. Continuous COC use can also be considered to prevent endometrioma recurrence after surgery, and to limit the risk of interfering with peak bone mass achievement in adolescents [200].

Dyspareunia generally responds less well to hormonal treatments because it is often caused by pressure exerted on deep lesions that infiltrate the Douglas structures and by stretching of dense adhesions during deep thrust. Progestins are indicated as first-line therapies here to achieve a sufficient anti-inflammatory action and avoid even minor lesion stimulation caused by the estrogens contained in COCs [111]. If these fail, surgery could be considered with the patient, clearly disclosing the relatively high likelihood of no, partial, or only temporary benefit, given that the nature of this symptom is often multifactorial. Progestins are also indicated for patients with non-subocclusive, deeply infiltrating bowel lesions, and for cases in which COCs are ineffective, not tolerated, or contraindicated.

Nonmenstrual, acyclic pelvic pain that does not respond to first-line medications should be treated with GnRH analogues to reduce nociceptive and, hypothetically, also nociplastic pain, via hypoestrogenism and inflammation switch-off.

4. Tolerability

A recent systematic review, including four RCTs and three observational studies, did not demonstrate an increased risk of psychological or sexual dysfunction in users of COCs and progestogens. Six studies showed a beneficial effect of hormonal therapy on dyspareunia. No treatment discontinuations due to psychological or sexual adverse events were reported if pain remission was achieved [201].

Despite these encouraging findings, depressed mood and reduced libido appear to affect a not negligible proportion of endometriosis patients who use progestogens or COCs, regardless of the overall effect on pain [202]. There are no clear biochemical explanations for these associations, and a causal relationship has not yet been definitively proven [203,204,205]. Theoretical endocrine properties offer little insight into the effects of different hormonal medications. A more sensible approach is to change medications until the one with the least impact on mood and libido for an individual patient is identified [155]. Before prescribing progestogen monotherapies or COCs, women should be specifically asked about previous or current psychiatric symptoms, diagnoses, and treatments. After providing adequate patient information and instruction on prompt discontinuation in case of worsening conditions during hormonal therapy, a careful shared decision should be made.

However, unexpected bleeding is the adverse effect that most influences tolerability [155]. Irregular bleeding is more than just an inconvenience; it contributes to persistent pelvic symptoms and plays an intricate role in the pathogenesis of pain, often affecting mood and fatigue as well. Irregular bleeding can and must be managed. The simplest approach is to instruct patients to practice “tailored bleeding”, which involves discontinuing progestogens or COCs for four to seven days whenever bleeding lasts for ≥5 days [155]. Administering a single dose of a depot GnRH agonist before starting progestogens or COCs has been suggested to reduce the incidence of unexpected bleeding [206]. Alternatively, a brief course of a GnRH agonist or antagonist can be prescribed if bleeding is not controlled by tailored cycling before resuming the use of first-line medications [155]. When the above measures fail, shifting to second-line compounds should be considered. For this condition, rather than an upfront combined approach, an add-on approach is suggested, i.e., the use of GnRH agonists or antagonists without add-back therapy for a few months to relieve irregular bleeding promptly and effectively. Such a measure should be undertaken to avoid prolonged suffering and reduced treatment adherence. Failing to do so can result in patients eventually refusing further hormonal therapies and requesting surgical procedures that could have otherwise been avoided.

5. Costs

GnRH agonists and antagonists are highly successful medications for women with symptomatic endometriosis, and their effectiveness appears to be superior to that of first-line medications [154]. Thus, one might wonder if starting with these drugs directly would not be the best option for patients. Unfortunately, GnRH analogues are very expensive, and prescribing them to all women with endometriosis would consume precious healthcare resources, considering the condition’s high prevalence and the need for prolonged treatment. The costs of therapy have an impact on individuals and their families and on the public health systems. A substantial proportion of patients forgoes care due to unaffordable costs [109]. Since at least two-thirds of patients with endometriosis are effectively treated by and satisfied with first-line medications [155], it would be ethically questionable not to adopt a stepped care approach starting with these safe, generally well-tolerated, and inexpensive therapeutics, and shifting to GnRH analogues only when earlier steps have been saturated.

6. Efficacy: What do we mean by “medical treatment failure”?

Two conditions must be met before declaring medical treatment failure: unexpected bleeding must be completely prevented or corrected, and GnRH analogs without add-back therapy must also be used unsuccessfully. Medical treatment encompasses more than just progestogens and COCs; all available pharmaceutical options must be tried before scheduling surgery or referring the patient to pain clinics [207,208]. Anovulation and amenorrhea are the foundation of hormonal therapy. If these conditions are not met, it may not be a matter of “failure”; rather, the problem may lie in suboptimal management by healthcare providers with limited experience using hormonal drugs correctly. Therefore, it is important to distinguish between real failure of hormonal therapy and failure to achieve either complete absence of bleeding or stable hypoestrogenism through a brief GnRH analog course without add-back therapy.

Until a few years ago, failure to respond to hormonal treatment was attributed to lesional progesterone resistance [209]. Now, non-response is largely attributed to nociplastic pain coexisting with nociceptive pain [29,30,31,37,179,180]. Both potential causes can be addressed by using GnRH analogs [209,210], as estrogens may facilitate central sensitization and pain hypersensitivity [211], especially within endometriosis-induced pelvic inflammatory status [4,212].

7. Secondary prevention: the earlier, the better

The prevalence of adenomyosis and endometriosis is very high among severely symptomatic adolescents and young women [55,213,214]. If left untreated, these conditions tend to progress [81,82,215]. In addition, a long delay in making a diagnosis and in starting suppressive treatment can facilitate the development of central sensitization. According to the guideline on CPP issued by the Society of Obstetricians and Gynaecologists Canada [40], “adolescents may be particularly susceptible to altered neural development, and thus adolescence may be a critical window for intervention to prevent the development of long-term pain morbidity in adulthood”. Therefore, severe dysmenorrhea and heavy menstrual bleeding should be promptly assessed, focusing specifically on the possible presence of early-onset adenomyosis and endometriosis. Timely hormonal and lifestyle interventions can improve HRQoL and limit the risk of nociplastic pain. In addition, adequately controlling adenomyosis and endometriosis prevents the progression of the disease to advanced forms that may require surgery, and preserves reproductive potential until a conception is desired. In essence, one of the main aims of hormonal treatment is to shift from treating the complicated forms, frequently associated with nociplastic pain in addition to nociceptive pain (i.e., tertiary prevention), to treating initial, not yet complicated forms causing only nociceptive pain (i.e., secondary prevention).

## 8. Conclusions

We have delineated an overall pharmacological strategy to manage endometriosis-associated pain symptoms in various and frequent conditions. The hormonal medications most frequently recommended in everyday practice are described in Table 1, where advantages and disadvantages of each drug category are summarized, along with a few practical hints based on both established evidence and clinical experience.

**Figure 1:**
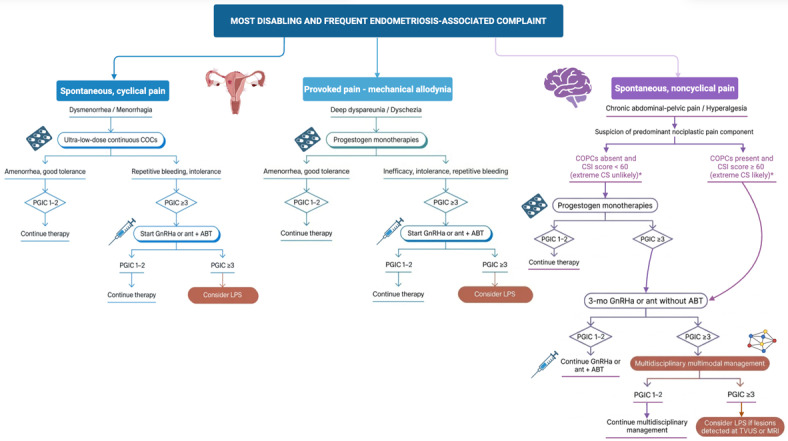
The proposed WMTY (What Matters To You) [199] treatment algorithm flowchart, based on self-reported outcome measures, for a symptom-oriented and patient-centered management of symptomatic endometriosis. It is intended for individuals who prefer pharmacologic menstrual suppression to surgery, can tolerate long-term hormonal treatment, and have no contraindications to it. ABT: add-back therapy. COCs: combined oral contraceptives. COPCs: chronic overlapping pain conditions. CS: central sensitization. CSI: 0 to 100-point central sensitization inventory [220]. GnRHa: GnRH agonist. GnRHant: GnRH antagonist. LPS: laparoscopy. MRI: magnetic resonance imaging. PGIC: 7-point patient global impression of change scale [216,217]. TVUS: Transvaginal Ultrasound. * Based on data from [224]. Created in BioRender. Bandini, V. (2026) https://BioRender.com/zs4cz4g.

In addition, a proposal for a stepwise treatment algorithm flowchart is shown in Figure 1. This clinical algorithm is envisaged exclusively for women with no absolute contraindications to COCs and progestogens. It is based on each patient’s priority for relief of the most distressing symptom. The flowchart is also tailored by the Patient Global Impression of Change (PGIC), a patient-reported outcome measure (PROM) that captures the individual assessments of global improvement and satisfaction with treatments [216] and is recommended by the Initiative on Methods, Measurement, and Pain Assessment in Clinical Trials [217]. Using the PGIC, patients are asked to rate their overall condition since treatment began on a 7-point scale (1 = very much improved; 2 = much improved; 3 = minimally improved; 4 = no change; 5 = minimally worse; 6 = much worse; 7 = very much worse). We consider ratings of 1 and 2 as “response” and ratings of 3 to 7 as “non-response”. The definition of “non-response” is applied after at least three months of unsuccessful treatment. We also consider a rating of 3 as a “non-response” because, in our opinion, “minimally improved” may not constitute a satisfactory outcome for patients with severe symptoms [218,219].

In the algorithm subsection focusing on patients experiencing mainly noncyclical chronic abdominopelvic pain and hyperalgesia, we included arbitrary cutoff scores based on our center’s experience [218,219] with the use of the Central Sensitization Inventory (CSI). The CSI is a questionnaire aimed at identifying pain complicated by central nervous system sensitization [220]. It has also been validated in patients with endometriosis [221,222,223]. The CSI consists of two parts. Part A contains 25 questions and yields a score ranging from 0 to 100. Scores between 60 and 100 indicate extreme central sensitization [224]. Part B assesses the presence of comorbid COCPs associated with central sensitivity dysfunctions. More information and details can be found in the reviews by Cetera et al. [219,225].

For patients with a CSI score > 60 points, or for those for whom progestogens are ineffective, a three-month course of a GnRH agonist or antagonist without add-back therapy aims to define the hormone-independent, and presumably mostly nociplastic, pain component [155,218,219,226]. This pharmacological test is readily feasible and may help identify the best candidates for additional multidisciplinary, multimodal interventions, such as an anti-inflammatory diet, aerobic exercise, pelvic floor rehabilitation, botulinum toxin application, acupuncture, and cognitive behavioral therapy [155,219]. In severely symptomatic patients with noncyclical chronic abdominal-pelvic complaints who do not respond to a GnRH analogue without add-back therapy, the question arises as to whether endometriosis is the sole or main trigger of pain, especially when deep infiltrating or ovarian lesions are not identified by TVUS or MRI [226].

The algorithm shown in Figure 1 is not comprehensive. It is experience-based, not evidence-based, and requires prospective validation. It only constitutes a general proposal for a symptom-oriented approach instead of a lesion-oriented approach. This pragmatic scheme, which is largely based on classical medical semeiology, can be used by healthcare providers with average experience treating endometriosis. It does not require sophisticated imaging techniques or an invasive diagnosis.

Available pharmacological therapies should be customized to select the safest, most effective, and best-tolerated individual option for each patient with adenomyosis and endometriosis. Because drugs come in packages with approved standard doses, gynecologists may believe that no special training is necessary to treat endometriosis-associated pelvic pain with hormones. However, we recommend that patients who do not respond to or tolerate first-line medications be referred to centers where they can be treated by healthcare providers with specific experience in the medical therapy of these diseases.

Finally, prospective cohort studies should be conducted to verify whether prolonged suppression of endometriosis results not only in a local pelvic, but also in a systemic anti-inflammatory effect, and reduces the risk of associated cardiovascular, autoimmune, and chronic inflammatory disorders [227,228,229].

## Figures and Tables

**Table 1:** Principal medications available for the hormonal management of patients with symptomatic adenomyosis/endometriosis and no absolute surgical indications or current pregnancy desire *.

Drug Class	Main Symptom and Indications	Type of Lesion	Pros	Cons	Tips & Tricks	Comment
Ultra-low-dose estrogen-progestogen combinations containing body-identical estrogens (i.e., estradiol or estetrol)	Dysmenorrhea as the most disabling symptom. Prevention of postoperative endometrioma recurrence. To be preferred over progestogen monotherapies in adolescents to avoid excessive interference with achievement of peak bone mass.	Superficial peritoneal endometriosis (low-risk lesions) and ovarian endometriomas (intermediate-risk lesions).	Popular, generally well-accepted and tolerated medications. Breakthrough bleeding frequent but easy to manage. Inexpensive. Substantial reduction in postoperative endometrioma and pain symptoms recurrence risk. Long-lasting reduction in ovarian, endometrial, and colon cancer risk.	Several but frequently limited side effects, including weight gain, bloating, depression, decreased libido. Risk of VTE associated with estrogen type (particularly increased when EE is used) and dose. Limited and temporary increase in breast-cancer risk.	Suggest a continuous flexible regimen, with 4 to 7-day hormone-free intervals triggered by breakthrough bleeding or prolonged spotting of ≥5 days, and followed by resumption of continuous COC use until the next bleeding episode	Start with oral preparations containing DNG or NOMAC, which generally ensure good bleeding control. Shift to combinations containing DRSP in case of weight gain, fluid retention, and psychological adverse effects. Avoid combinations containing EE.
Oral progestogens	Deep dyspareunia as the most distressing symptom. Contraindications or intolerance to estrogen-progestogen combinations.	Deep infiltrating endometriosis (high-risk lesions).	Safe and generally well tolerated. DNG, DRSP, and NETA can be used in most patients with contraindications to COCs, including frequent headaches and migraine with aura. Inexpensive.	Progestogens can exacerbate depression, decrease libido, and, except NETA and other androgenic compounds, reduce bone mineral content. Frequent breakthrough bleeding, which are more difficult to manage if subdermal implants are used. Avoid long-term CPA use due to the considerable increase in meningioma risk. DMPA associated with increased VTE risk and bone demineralization.	NETA can be used at a 2.5 mg/daily dose to limit incidence of androgenic-type adverse effects and VTE risk. Inform patients how to manage bleeding episodes (tailored cycling). Shift to DRSP if weight gain, depression, or reduced libido with DNG. Shift to DNG if excessive bleeding with DRSP.	Oral progestogens can be combined with transdermal estradiol gel or patches to limit hypoestrogenic symptoms and bone demineralization. Cholecalciferol (vitamin D3), 1000–1200 IU/day plus calcium, ≈1000 mg/day, can limit bone mass loss.
LNG-releasing intrauterine devices	Dysmenorrhea and menorrhagia as the most disabling symptoms.	Adenomyosis (all forms).	Safe, generally well tolerated. Can be used in patients with contraindications to COCs. Inexpensive when used for the entire period of LNG-IUD efficacy. Provides excellent contraception. No need for daily tablet use.	Insertion potentially painful, especially with the larger 52 mg device. Limited protection from ovarian endometrioma development/recurrence as ovulation is not suppressed. Androgenic-type adverse effects and depression exacerbation due to LNG absorption. Unfeasible option before sexual debut.	The use of the 52 mg LNG-IUD is indicated in adult or parous women and when moderate to severe adenomyosis forms are detected. The 19.5 mg LNG-IUD may be preferred in sexually active adolescents with mild adenomyosis forms to limit the risk of untoward effects and facilitate insertion.	Insertion of an LNG-IUD can be considered after completion of a laparoscopic procedure for endometriosis in patients not seeking a postoperative conception and declining oral first-line drugs.
GnRH agonists	Non-response, intolerance, or contraindications to first-line medications. Noncyclical chronic pelvic pain as the most disabling symptom. Coexistence of chronic overlapping pain conditions. High clinical suspicion of a considerable nociplastic component.	All phenotypes, but particularly useful for symptoms associated with deep infiltrating lesions not controlled by progestogen monotherapies. Amenorrhea is generally achieved also in patients with severe adenomyosis.	GnRH agonists are highly effective and can control pain symptoms when first-line medications fail. A short GnRH agonist course without ABT can be used ex juvantibus to discriminate between estrogen-dependent and estrogen-independent pain and discern whether endometriosis is the main source of pain.	Estrogen deprivation symptoms. Progressive reduction in bone mineral content during prolonged use without ABT. Potential financial toxicity due to high drug cost.	Stepping up to GnRH analogues should not be delayed when first-line medications are ineffective, not tolerated, or cause unmanageable, repeated breakthrough bleeding. Injecting triptorelin 3.75 mg every six instead of four weeks, and 11.25 mg every 16 instead of 12 weeks, allows a substantial cost reduction without loss of efficacy. To avoid flare-up, administer the first depot injection at midluteal phase or during a 10-day oral progestogen course.	Timing, type, dose, and delivery route of ABT can be customized. Depot GnRH formulations can be combined with transdermal estradiol gel or patches, plus oral or vaginal progesterone, 200 mg/day; or with a weekly combined transdermal patch releasing estradiol, 50 μg and LNG, 7 μg/24 h; or with oral tibolone, 2.5 mg/day. ABT should be temporary discontinued in case of breakthrough bleeding.
GnRH antagonists	Non-response, intolerance, or contraindications to first-line medications. Non-cyclic chronic pelvic pain as the most disabling symptom. Coexistence of chronic overlapping pain conditions. High clinical suspicion of a considerable nociplastic component. Similarly to GnRH agonists, also GnRH antagonists can be briefly used ex juvantibus, without ABT, to discern whether endometriosis is the main source of pain.	All phenotypes, but particularly useful for symptoms associated with deep infiltrating lesions not controlled by progestogen monotherapies. Amenorrhea is generally achieved also in patients with severe adenomyosis.	GnRH antagonists at high doses are similarly highly effective as GnRH agonists and can control pain symptoms when first-line medications fail. The oral rather than the parenteral route is preferred by some patients. The medication can be immediately discontinued if adverse effects ensue. The GnRH antagonists can be combined with ABT in a single tablet. However, this may nor reveal opportune when ABT customization is desired or when its temporary discontinuation is required because of unexpected, prolonged bleeding episodes.	Low-dose GnRH antagonists suppress estradiol synthesis only partially and ABT can be avoided. However, low-dose GnRH antagonists are associated with more frequent erratic bleeding, and suboptimal pain relief.	Low-dose GnRH antagonists do not consistently suppress ovulation. When using low-dose regimens, serial pregnancy tests would be required to promptly differentiate between pregnancy and drug-induced amenorrhea. Full-dose regimens combined with ABT in the same tablet appear practical but prevent treatment customization. Moreover, a separate ABT (i.e., not included in the same tablet) allows its temporary discontinuation in case of breakthrough bleeding.	GnRH antagonists are as expensive as GnRH agonists. This can limit access to therapy, reduce treatment adherence, and result in disparities in countries without a publicly funded healthcare system.

* Modified from Table 2 included in [155] and generally based on recommendations from the ESHRE guideline [38], the updated NICE guideline [39], and the SOGC guideline [40] for managing endometriosis and chronic pelvic pain (CPP). ABT, add-back therapy; COC, combined oral contraceptive; CPA, cyproterone acetate; DMPA, depot-medroxyprogesterone acetate; DNG, dienogest; DRSP, drospirenone; EE, ethinyl estradiol; GnRH, gonadotropin-releasing hormone; LNG-IUD, levonorgestrel-releasing intra-uterine device; NETA, norethisterone acetate; NOMAC, nomegestrol acetate; VTE, venous thromboembolism.

## Data Availability

No new data were created or analyzed in this study.
